# Time-dependent suicide rates among Army soldiers returning from an Afghanistan/Iraq deployment, by military rank and component

**DOI:** 10.1186/s40621-022-00410-9

**Published:** 2022-12-23

**Authors:** Rachel Sayko Adams, Jeri E. Forster, Jaimie L. Gradus, Claire A. Hoffmire, Trisha A. Hostetter, Mary Jo Larson, Colin G. Walsh, Lisa A. Brenner

**Affiliations:** 1 Department of Health Law, Policy and Management, Boston University School of Public Health, 715 Albany Street, Boston, MA 02118 USA; 2 Institute for Behavioral Health, The Heller School for Social Policy and Management, Brandeis University, Waltham, MA USA; 3 VHA Rocky Mountain Mental Illness Research Education and Clinical Center, Aurora, CO USA; 4 University of Colorado, Anschutz Medical Campus, Aurora, CO USA; 5 Department of Epidemiology, Boston University School of Public Health, Boston University, Boston, MA USA; 6 Departments of Biomedical Informatics, Medicine, and Psychiatry, Vanderbilt University Medical Center, Nashville, TN USA

**Keywords:** Suicide, Military, Veterans, Deployment

## Abstract

**Background:**

To date, knowledge is limited regarding time-dependent suicide risk in the years following return from deployment and whether such rates vary by military rank (i.e., enlisted, officer) or component (i.e., active duty, National Guard, reserve). To address these gaps in knowledge, the objectives of this study were to determine and compare postdeployment suicide rates and trends (percent change over time), and hazard rates for Army soldiers, by rank and component (measured at the end of the deployment).

**Methods:**

Longitudinal cohort study of 860,930 Army soldiers returning from Afghanistan/Iraq deployment in fiscal years 2008–2014 from the Substance Use and Psychological Injury Combat study. Death by suicide was observed from the end of the first deployment in the study period through 2018 (i.e., the most recently available mortality data) for up to 11 years of follow-up. Analyses were conducted in 2021–2022.

**Results:**

Adjusting for age, lowest-ranking Junior Enlisted (E1–E4) soldiers had a suicide rate 1.58 times higher than Senior Enlisted (E5–E9)/Warrant Officers (95% CI [1.24, 2.01]) and 2.41 times higher than Officers (95% CI [1.78, 3.29]). Suicide rates among lower-ranking enlisted soldiers remained elevated for 11 years postdeployment. Overall and annual postdeployment suicide rates did not differ significantly across components. Comparisons across rank and component for females were generally consistent with the full cohort results.

**Conclusions:**

Lower-ranking enlisted soldiers had the highest rate of suicide, underscoring the importance of understanding rank as it relates to social determinants of health. For over a decade following Afghanistan/Iraq deployment, lower-enlisted rank during deployment was associated with an elevated rate of suicide; thereby suggesting that postdeployment prevention interventions targeting lower-ranking military members are warranted.

**Supplementary Information:**

The online version contains supplementary material available at 10.1186/s40621-022-00410-9.

## Background

Deployment is an experience unique to military members that may increase suicide risk (Shen et al. 2016; Schoenbaum et al. 2014). Deployments to Afghanistan and Iraq have been associated with combat exposure (Bryan et al. 2015; LeardMann et al. 2021) and physical and mental health conditions (Psychological Health Center of Excellence Research and Development Directorate 2019; Hostetter et al. 2019) which have been demonstrated to increase suicide risk. While prior research has shown that both military rank (e.g., enlisted versus officers) and component (active duty [AD], National Guard [NG], reserve component [RC]) are associated with suicide risk (Ravindran et al. 2020), knowledge is limited regarding time-dependent suicide risk in the years following return from deployment and whether such rates vary by rank or component.

Furthermore, postdeployment suicide risk may differ for military members returning from their first deployment versus those returning from subsequent deployments. Investigations have identified a “healthy warrior effect” such that less psychologically fit military members leave military service sooner (Larson et al. 2008). There is speculation that this phenomenon continues after each deployment, thus more “healthy” and resilient members are available for future deployments (Larson et al. 2013). A study of AD Army soldiers who died by suicide during military service between 2004 and 2009, found the highest suicide rate was among lower-ranking Enlisted soldiers deployed during their first year of service (Gilman et al. 2014). Yet, multiple deployments inherently increase potential for combat exposure, which may increase suicide risk (Bryan et al. 2015).

According to annual Department of Defense (DoD) suicide surveillance and studies during the Afghanistan/Iraq conflicts, lowest-ranking Junior Enlisted (E1–E4) members were at highest suicide risk compared to Senior Enlisted or Officers (Psychological Health Center of Excellence Research and Development Directorate 2019 Annual Report 2019; Pruitt et al. 2016; Hyman et al. 2012). Military rank is a defining characteristic of service that influences the military experience in predictable ways, including training, job expectations, and deployment exposures. Rank is associated with social determinants of health (e.g., socioeconomic status, race/ethnicity) (Department of Defense 2020) that may influence suicide risk (Martínez-Alés et al. 2021). During the Afghanistan/Iraq conflicts, people from less wealthy communities, with fewer educational and professional opportunities disproportionately served in the military (US Army Recruiting Command - Official Website 2022). Such individuals often joined after high school, and entered service at the Junior Enlisted rank (Rostker et al. 2014).

Military component also reflects differences in military experience that may influence postdeployment health and suicide risk. DoD suicide surveillance reports have shown that AD members had the highest suicide risk compared to NG/RC members, yet reasons are unclear (Psychological Health Center of Excellence Research and Development Directorate 2019 Annual Report 2019; Pruitt et al. 2016) AD members have full-time service commitments (usually 2–6 years), typically live on military bases, and can be deployed at any time (Veterans Affairs National Center for PTSD 2012). NG and RC members generally serve part-time while living at home, report for training one weekend a month and 2 weeks a year, and can be called upon to deploy (Veterans Affairs National Center for PTSD 2012; Military.com. 2022). Postdeployment, AD members typically return to live on military bases. NG/RC members generally return home postdeployment and hold civilian jobs; in turn, they may have less connection to military culture and monitoring from Commanders (Adams et al. 2020).

To better understand time-dependent postdeployment suicide risk by military rank and component, this study examined a population-based cohort of 860,930 soldiers returning from an Afghanistan/Iraq deployment between fiscal years (FYs) 2008–2014 (the first deployment in this period was defined as the index deployment). Study objectives were to: (1) estimate average annual and time-dependent suicide rates and trends by rank and component; (2) estimate the relative hazard for suicide and compare suicide rates and trends across rank and component; (3) estimate and compare suicide rates and estimate the relative hazard for suicide between those returning from a first deployment versus those with multiple deployments, by rank and component; and (4) determine if results were different among female soldiers compared to the full cohort. For all objectives, rank and component were measured at the end of the index deployment. Knowledge generated may provide novel information about time-dependent suicide risk during the postdeployment window specific to rank and component, with the goal of preventing military and Veteran suicide.

## Methods

### Data sources

Study data were drawn from the Substance Use and Psychological Injury Combat study (SUPIC), an observational, population-based, longitudinal cohort study of all Army soldiers returning from an Afghanistan/Iraq deployment which ended in FYs 2008–2014 (Larson et al. 2013). Deployment data were obtained from the DoD’s Contingency Tracking System maintained by the Defense Manpower Data Center to identify the index Afghanistan/Iraq deployment (i.e., first deployment associated with the Afghanistan/Iraq conflicts ending within the study window), and Afghanistan/Iraq deployment history prior to the index deployment dating back to the start of the conflicts in Afghanistan. Other deployments to non-contingency operations (i.e., peace-keeping missions) were not included in these data. Demographic characteristics were from DoD’s Defense Enrollment Eligibility Records System. Mortality data were obtained from the VA/DoD mortality data repository (MDR), which contains all-cause mortality data from the National Death Index (NDI), consisting of death record data from state vital statistics offices.

### Study population

Using the SUPIC finder file (*n* = 865,640 soldiers), soldiers were excluded if they did not have a usable social security number (SSN; *n* = 141), required to search the NDI through the MDR. An additional 1123 soldiers were excluded because their NDI-documented death date was before the end of their index deployment. After these exclusions were applied, inclusion criteria for being in the analytic cohort included: (1) having military component data at the end of index deployment; (2) having an index deployment lasting ≤ 5 years; and (3) ensuring SSN and demographic data consistency within and between the SUPIC finder file, Veterans Health Administration medical record data, and the MDR (i.e., date of birth, gender). The application of these exclusion and inclusion criteria resulted in a final analytic cohort of 860,930 (99.5% of original SUPIC file; Additional file 1).

### Measures

Death by suicide, the study outcome, was determined by identifying records within the NDI containing International Classification of Diseases, Tenth Revision (ICD-10) codes X60-X84 and Y87.0 as the underlying cause of death. Independent variables of interest included rank (Junior Enlisted [E1–E4], Senior Enlisted [E5–E9]/Warrant Officer, and Officer), and component (AD, NG, and RC). Deployment group was defined as first deployers (i.e., the index Afghanistan/Iraq deployment that ended in the study window was the first deployment) or 2 + deployers (i.e., the index deployment that ended in the study window was their second or more deployment associated with the Afghanistan/Iraq conflicts). Covariates included age (18–24, 25–29, 30–34, 35–39, 40 +), gender, race/ethnicity (White non-Hispanic, Black non-Hispanic, Hispanic, Asian American or Pacific Islander, American Indian/Alaskan Native, other/unknown), and FY return from index deployment (2008–2009, 2010–2011, 2012–2014).

### Statistical analysis

Demographic and military characteristics were summarized for: (1) the overall analytic cohort; (2) by component; (3) by deployment group; and (4) by component within deployment group, using frequencies. The follow-up period for death by suicide was from October 1, 2007 – December 31, 2018 (range 0–11 years postdeployment). Crude and age-adjusted average annual suicide rates (using the direct standardization method) were calculated per 100,000 person-years over the follow-up period within rank groups, deployment groups and by component. Age-adjusted rates were standardized based on the 2000 US population (Klein 2000), using age categories 18–24, 25–29, 30–34, 35–39, 40 + . In some cases, it was necessary to collapse age categories in order to achieve age-adjustment due to small event sizes (i.e., number of suicides in a subgroup). Rates based on < 16 suicides were denoted as unreliable, and < 10 were suppressed (Division of Cancer Prevention and Control - Centers for Disease Control and Prevention 2021). Rate ratios were computed, when feasible, to compare age-adjusted rates. Rates and rate ratios were presented with 95% confidence intervals (CIs). As suicide was a rare event, crude rates were presented with exact CIs, age-adjusted rates were presented with CIs based on the gamma distribution given its improved coverage over other methods (Fay and Feuer 1997), and rate ratios with CIs based on the inverse of the *F* distribution (Fay 1999).

Suicide rates since return from index deployment were calculated for 1- or 2-year intervals (depending on cell sizes) and presented per 100,000 person years—within rank group and component. Annual percent change (APC) in suicide rates was estimated using trend analysis (joinpoint regression) (Kim et al. 2000), and differences in trends (slopes) were compared using tests for parallelism (Kim et al. 2004) (comparison of joinpoint regression models). Cox proportional hazard models were performed to separately evaluate the relationship between each independent variable of interest and suicide risk. Models were run unadjusted and adjusted for age category, gender, race/ethnicity, and FY of return from index deployment. Consistent with the modern epidemiologic definition of confounders, variables included for adjustment: (1) could not be on the causal pathway between rank and suicide (e.g., psychiatric disorders occurring in this time period), (2) were unevenly distributed by rank, and (3) were independently associated with suicide (VanderWeele et al. 2021). Age and race/ethnicity categories for adjustment were collapsed when necessary due to small cell sizes. Hazard ratios (HR) are presented with 95% CIs. The proportional hazards assumption was met for all variables and was assessed using the ZPH (weighted Schoenfeld residuals) and supremum test diagnostics.

Statistical significance was assessed using an alpha of 0.05. Beyond statistical significance, effect sizes and patterns of findings were evaluated. Analyses were performed in SAS software v9.4 (SAS Institute, Cary NC; Cox proportional hazards models), R v4.1.1 (R Core Team 2000) (suicide rates, trend graphs) and Joinpoint (National Cancer Institute 2022) (trend analyses and parallelism tests). Analyses were conducted in 2021–2022.

## Results

Most of the cohort was aged 18–29 at the end of their index deployment (62.4%), male (89%) and White non-Hispanic (62.7%—Additional file 2). Two-thirds were AD, 24.0% was NG and 9.4% was RC. Junior Enlisted was the most prevalent rank among AD and NG members. Among RC, approximately one-third were Junior Enlisted, while 44.5% were Senior Enlisted/Warrant Officer. The index deployment was the first deployment for 69.5% of the cohort (Additional file 3). Distributions of age and rank within deployment group and component were similar between the full sample and the female sample (Additional file 4).

### Rank analysis

Adjusting for age, Junior Enlisted members had a suicide rate 1.58 times that of Senior Enlisted/Warrant Officers (95% CI [1.24, 2.01]) and 2.41 times that of Officers (95% CI [1.78, 3.29]). Senior Enlisted/Warrant Officers had an age-adjusted rate 1.53 times that of the Officers (95% CI [1.22, 1.92]). When stratifying by deployment group, findings remained largely consistent with regards to both effect size and statistical significance (Table 1).

**Table 1:** Average annual suicide rates per 100,000 person years (October 1, 2007–December 31, 2018)

Rank	Crude rate (95% CI)	Age-adjusted rate^a^ (95% CI)	Rate ratio (95% CI)
Junior Enlisted (E1–E4)	49.84 (47.48, 52.29)	38.23 (30.64, 47.63)	vs. SE/WO: **1.58 (1.24, 2.01)** vs. Officers: **2.41 (1.78, 3.29)**
Senior Enlisted (E5–E 9)/Warrant Officers	29.62 (27.69, 31.65)	24.18 (22.04, 26.52)	vs. Officers: **1.53 (1.22, 1.92)**
Officers	16.31 (13.78, 19.17)	15.83 (12.84, 19.38)	Ref
Rank Among First Deployers			
Junior Enlisted (E1–E 4)	50.41 (47.87, 53.05)	39.15 (30.84, 49.61)	vs. SE/WO: **1.66 (1.25, 2.17)** vs. Officers: **2.42 (1.69, 3.50)**
Senior Enlisted (E5–E9)/Warrant Officers	28.50 (25.75, 31.46)	23.65 (20.73, 26.95)	vs. Officers: **1.46 (1.09, 1.98)**
Officers	16.12 (13.03, 19.73)	16.20 (12.32, 21.04)	Ref
Rank Among 2 + Deployers			
Junior Enlisted (E1–E 4)	46.02 (39.88, 52.85)	33.48^b^ (24.53, 45.05)	vs. SE/WO: 1.20 (0.87, 1.65) vs. Officers: **2.01 (1.32, 3.07)**
Senior Enlisted (E5–E 9)/Warrant Officers	30.60 (27.93, 33.45)	27.82^b^ (25.17, 30.69)	vs. Officers: **1.67 (1.25, 2.27)**
Officers	16.67 (12.49, 21.81)	16.67^b^ (12.48, 21.87)	Ref

*SE* Senior Enlisted, *WO* Warrant Officers, *Ref* reference group

^a^Age-adjusted rates based on age categories 18–24, 25–29, 30–34, 35–39, 40 +

^b^Age-adjusted rates based on age categories 18–29 and 30 +

Boldface indicates statistical significance (*p* < 0.05)

**Fig. 1:**
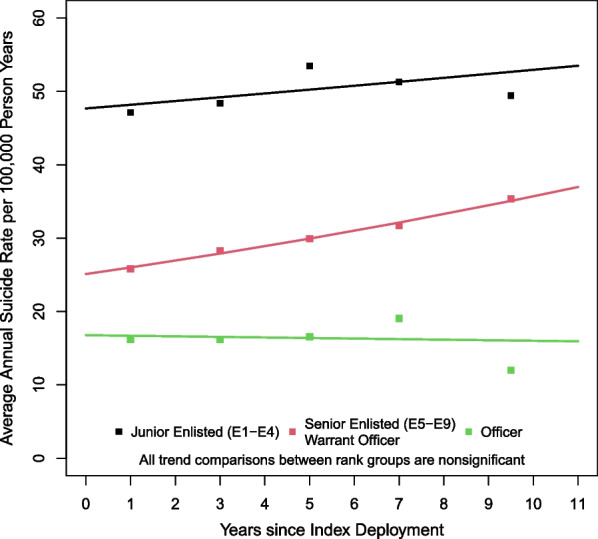
Rank average annual suicide rates per 100,000 person years with trend lines

Junior Enlisted members had the highest average annual suicide rates, which remained elevated through 11 years postdeployment and above the estimated suicide rates for Senior Enlisted/Warrant Officers and Officers, who had the lowest rates (Table 2). The trend analysis revealed a significant trend for Senior Enlisted/Warrant Officers with an estimated average annual increase of 3.6% (95% CI [3.0%, 4.2%]) and while the estimated trend for Senior Enlisted/Warrant Officers and Officers were lower (1.1% and − 0.5%, respectively; Table 2), no significant differences were observed in pairwise tests for parallelism (i.e., evidence was insufficient to indicate trends differed over time across rank groups; Table 2 and Fig. 1).

**Table 2:** Average annual suicide rates since end of index deployment, by rank, with 95% confidence intervals

Rank full cohort	Years since end of index deployment					
	0–2 years	2–4 years	4–6 years	6–8 years	8–11 years	APC^a^ (95% CI)
Junior Enlisted (E1–E4)	47.12 (42.4, 51.8)	48.37 (43.6, 53.1)	53.48 (48.3, 58.6)	51.29 (45.5, 57.1)	49.42 (41.9, 56.9)	1.1 (− 1.7, 3.8)
Senior Enlisted (E5–E9)/Warrant Officer	25.83 (22.0, 29.7)	28.25 (24.2, 32.3)	29.92 (25.7, 34.1)	31.72 (27.1, 36.4)	35.34 (29.4, 41.3)	**3.6 (3.0, 4.2)**
Officer	16.18 (10.8, 21.5)	16.20 (10.8, 21.6)	16.57 (11.0, 22.1)	19.04 (12.3, 25.7)	11.99 (5.2, 18.8)	− 0.5 (− 8.0, 7.7)

Suicide rates are per 100,000 person years

Boldface indicates statistical significance (*p* < 0.05)

^a^Annual percent change estimated using trend analysis

Among the full cohort, Junior Enlisted members had an increased unadjusted hazard for death by suicide as compared to Senior Enlisted/Warrant Officers and Officers; Senior Enlisted/Warrant Officers had a higher unadjusted hazard than Officers (Table 3). These associations were slightly attenuated but remained significant in the model adjusted for demographics. The adjusted hazard of death by suicide for Junior Enlisted members relative to Senior Enlisted/Warrant Officers was 1.35 (95% CI 1.23, 1.49) and relative to Officers was 2.38 (95% CI 1.99, 2.85). The adjusted hazard of suicide for Senior Enlisted/Warrant Officers relative to Officers was 1.76 (95% CI 1.48, 2.10).

**Table 3:** Hazard ratios from unadjusted and adjusted Cox proportional hazard models

Full cohort	Unadjusted model	Adjusting for Demographics^a^
	Hazard Ratio (95% CI)	Hazard Ratio (95% CI)
Junior Enlisted (E1–E4) versus Senior Enlisted (E5-E9)/Warrant Officer	**1.69** **(1.55, 1.83)**	**1.35** **(1.23, 1.49)**
Junior Enlisted (E1–E4) versus Officer	**3.06** **(2.58, 3.62)**	**2.38** **(1.99, 2.85)**
Senior Enlisted (E5–E9)/Warrant Officer versus Officer	**1.81** **(1.52, 2.16)**	**1.76** **(1.48, 2.10)**
First Deployers		
Junior Enlisted (E1–E4) versus Senior Enlisted (E5–E9) /Warrant Officer	**1.77** **(1.59, 1.98)**	**1.45** **(1.28, 1.66)**
Junior Enlisted (E1–E4) versus Officer	**3.13** **(2.54, 3.85)**	**2.64** **(2.12, 3.29)**
Senior Enlisted (E5–E9)/Warrant Officer versus Officer	**1.76** **(1.48, 2.21)**	**1.82** **(1.45, 2.28)**
2 + Deployers		
Junior Enlisted (E1–E4) versus Senior Enlisted (E5–E9) /Warrant Officer	**1.50** **(1.28, 1.77)**	1.17 (0.98, 1.40)
Junior Enlisted (E1–E4) versus Officer	**2.75** **(2.04, 3.73)**	**1.84** **(1.33, 2.55)**
Senior Enlisted (E5–E9)/Warrant Officer versus Officer	**1.83** **(1.38, 2.43)**	**1.57** **(1.18, 2.10)**

^a^Adjusted for gender, age category (18–24, 25–29, 30–34, 35–39, 40 +), race/ethnicity (American Indian/Alaskan Native, Asian or Pacific Islander, Black non-Hispanic, White non-Hispanic, Hispanic, other, unknown/missing) and Fiscal Year of return from index deployment grouped as 2008–2009, 2010–2011, and 2012–2014

Boldface indicates statistical significance (*p* < 0.05)

Results among first deployers were similar to the full cohort results. When assessing the hazard for suicide by rank among 2 + deployers, unadjusted results were like the full cohort and first deployer models, with slightly lower hazard for Junior Enlisted relative to both Senior Enlisted/Warrant Officers and Officers. In the adjusted 2 + deployer model, Junior Enlisted had a significantly higher hazard for suicide compared to Officers (HR = 1.84; 95% CI 1.33, 2.55), and Senior Enlisted/Warrant Officers had a higher hazard compared to Officers (HR = 1.57; 95% CI 1.18, 2.10). The overall pattern revealed a larger magnitude of increased risk for Junior Enlisted and Senior Enlisted/Warrant Officers among first deployers than 2 + deployers.

### Component analysis

Suicide rates and unadjusted and adjusted hazard for suicide did not differ significantly across components (Additional files 5 and 6) with the exception that among 2 + deployers, RC has a lower age-adjusted suicide rate relative to AD (Rate Ratio = 0.68, 95% CI 0.46, 0.97). Trends in suicide rates since return from index deployment did not significantly differ across component. AD did have an increase in suicide rates through 6.5 years post index deployment (APC = 3.9%, 95% CI 1.3%, 6.6%), followed by a nonsignificant decrease from 6.5 to 11 years (APC = − 3.5%, 95% CI − 10.9%, 4.5%), while NG and RC each had a small increase and decrease over time, respectively (Additional files 7 and 8).

### Models for female soldiers

We conducted analyses among female soldiers, as feasible, to determine if associations between suicide and (1) rank and (2) component for females were similar to those for the full cohort. Females comprise 11% of the SUPIC cohort and had a significantly lower age-adjusted suicide rate than their male counterparts (10.59 vs. 27.61, respectively). Females were 62% less likely to die by suicide than male soldiers (95% CI − 73%, − 45%; age-adjusted rate ratio: 0.38; 95% CI 0.27, 0.55). Given the combination of a lower overall sample size and a lower suicide rate, the number of events was small and limited the analyses that could be conducted in the female sample. As such, only the unadjusted and adjusted Cox proportional hazard models were run. Overall, estimates were less precise for the female models than those for the full cohort and mostly nonsignificant. However, the estimated hazard ratios were consistent in direction of association (i.e., above or below 1) between the female and full cohort models with the exception of the adjusted comparisons between Junior Enlisted and Senior Enlisted/Warrant Officers and the unadjusted comparison between Junior Enlisted and Senior Enlisted/Warrant Officers for 2 + deployers (Additional files 9 and 10). Additionally, while consistent in the direction of association, estimated hazard ratios were larger among the female cohort for Senior Enlisted/Warrant Officers compared to Officers, overall and across deployment groups, than was observed among the full cohort. Conversely, the hazard ratios were smaller for Junior Enlisted compared to Officers in the female cohort as compared to the full cohort.

## Discussion

Suicide rates among military members and Veterans remain higher than among civilians (Hoge and Ivany 2017; Department of Veterans Affairs 2021; Pruitt et al. 2019). We found that for up to 11 years following return from an Afghanistan/Iraq deployment ending in FYs 2008–2014, Junior Enlisted (E1–E4) Army soldiers had the highest age-adjusted suicide rates and highest adjusted hazards for suicide. Senior Enlisted/Warrant Officers followed the same pattern of increased suicide risk compared to Officers. Thus, lower rank was consistently associated with elevated risk for suicide for over a decade following deployment. These findings are critical as Junior Enlisted represent over half of the enlisted workforce, which represents 82% of the Armed Forces (Congressional Research Service 2021). An innovation of this study was our ability to examine trends in suicide rates prospectively postdeployment. Although we only observed a significant average annual increase in suicide of 3.4% among Senior Enlisted/Warrant Officers, there was not sufficient evidence to conclude that trends over time differed across the rank groups. Thus, the observed elevated postdeployment suicide rates among soldiers, particularly those of lower-enlisted rank, started and remained elevated for many years postdeployment.

Rank is associated with other social determinants of health which themselves are associated with suicide risk (e.g., socioeconomic status, race/ethnicity) (Department of Defense 2020; Martínez-Alés et al. 2021). Rank influences paygrade, with Junior Enlisted earning the lowest pay compared to Senior Enlisted, Warrant Officers and Officers (Defense Finance and Accounting Service 2021). Financial strain and lower socioeconomic status increase suicide risk (Martínez-Alés et al. 2021; Elbogen et al. 2020). Racial inequities exist in the military including disparities in military leadership and rank. In 2020, half of the lowest rank positions (E1–E2) were filled by minority military members, compared to only 10% of the highest ranks (O9–O10) (Department of Defense 2020). Thus, reduced opportunities and earning potential of Junior Enlisted members are disproportionally held by minority members. Additionally, while suicide rates among Army soldiers follow similar patterns to civilian populations with non-Hispanic white and American Indian/Alaskan Natives at highest risk, suicide rates tend to be higher among all racial/ethnic groups compared to their civilian counterparts (Kochanek et al. 2016; Griffin et al. 2020). Taken together, military rank may itself be a social determinant of health that influences suicide risk.

We did not find variation in postdeployment suicide risk by component, unlike prior cross-sectional studies and surveillance reports which have found that AD members have higher suicide rates (Psychological Health Center of Excellence Research and Development Directorate 2019; Ravindran et al. 2020). When examining postdeployment suicide trends by component, AD members had a significant increase in hazard rates through 6.5 years postdeployment, with no significant trends over time observed for NG or RC. The period immediately after leaving military service is associated with increased suicide risk (Shen et al. 2016); however, our analyses did not capture if and when separation from service occurred during the observation window. Our findings suggest that for AD members, the first 6 years postdeployment is a period of increasing suicide risk, which likely captures military separation for many, and may imply reintegrating into civilian life after living on a military base creates additional risk. Future research is warranted to determine if trends in suicide rates vary over time by component (or rank) post-military service.

Over two-thirds of our sample was returning from a first deployment. Some have speculated that military members with multiple deployments may represent “healthy warriors” who exhibit greater resilience and the ability to stay in the military and deploy again (Larson et al. 2008, 2013) The counter hypothesis is that additional deployments may increase cumulative burden of combat exposure and other trauma which may increase suicide risk. We found a larger magnitude of increased suicide rates for Junior Enlisted and Senior Enlisted/Warrant Officers among first deployers than 2 + deployers. This suggests some evidence for the “healthy warrior effect” in that these findings were attenuated among 2 + deployers. However, suicide rates remained elevated among soldiers in lower-ranking groups regardless of whether returning from a first or 2 + deployment; therefore, we should not assume that members who deploy multiple times are inherently protected from suicide risk, particularly when they are individuals of lower rank.

In the US, suicide rates among female military members/Veterans have been increasing over the past two decades (U.S. Department of Veterans Affairs^2021^, ^2016^). Females represented 11% of our study sample with the majority AD (65%) first deployers (78%) and Junior Enlisted (43%). Females represented only 4.3% of the number of deaths by suicide in the cohort, with a suicide rate of 14.72. Thus, our ability to make precise estimates and comparisons was reduced due to the small number of events. However, hazard ratios for females were generally consistent (i.e., had a similar direction of association, above or below 1) with the full cohort models. Female military members/Veterans should not be overlooked as suicide prevention programs are employed for the predominantly male military/Veteran population. Gender-sensitive initiatives should be developed or refined to address unique stressors and risk factors faced by female military members/Veterans (e.g., military sexual trauma, reintegration challenges) (Street et al. 2009; Adams et al. 2016).

## Limitations

Suicide is sometimes not coded when intent cannot be determined. We were unable to present statistical findings for some groups (e.g., Junior Officers, Warrant Officers) due to small samples sizes, particularly in the female and 2 + deployer models. We were unable to adjust for or make additional comparisons by race and ethnicity in this paper, due to sample size limitations when examining the associations between rank and component and suicide risk. Additional analyses are underway with this cohort to examine the association between race and ethnicity and suicide risk following index deployment, though these analyses do not evaluate rank and component differences simultaneously due to same sample size limitations. Our study included soldiers returning from Afghanistan/Iraq deployments during FYs 2008–2014, and these findings may not generalize to other branches of service or other years of the Afghanistan/Iraq conflicts. While military rank/paygrade is a good proxy for the income of military members at the time of return from their index deployment, we did not have additional information pertinent to the socioeconomic status of soldiers in this study (e.g., spousal income). We did not have access to Military Occupational Status information for the SUPIC cohort (e.g., infantry, healthcare), which may be associated with different military exposures during deployment and has been shown to be associated with suicide risk (Kessler et al. 2015). The goal of this study was to assess the effect of rank and component on postdeployment suicide rates. Psychological and substance use problems, and traumatic brain injury, increase suicide risk among military members/Veterans (Hostetter et al. 2019; Pruitt et al. 2019; Millner et al. 2019); however our data for these diagnoses were only available during the postdeployment window. Therefore, these variables cannot be confounders of the association between rank or component and suicide because they do not precede rank or component in time and we did not adjust for these variables (VanderWeele et al. 2021). Psychological, substance use problems or traumatic brain injury may be on the causal pathway from rank or component to suicide and therefore may act as mediators of the association we observed between rank and suicide. This is an important area for future research.

## Conclusions

This study captures death by suicide among a population-based cohort of 860,930 Army soldiers returning from an Afghanistan/Iraq deployment during a 6-year period of intense military operations from the deployment return through 2018. Lower-ranking enlisted soldiers (E1–E4) were at highest risk for suicide in the years following return from deployment, highlighting the importance of understanding rank as a potential social determinant of health which may increase postdeployment suicide risk. As lower-rank was associated with elevated risk for suicide for many years following deployment, prevention interventions targeting lower-ranking military members/Veterans following deployment may be required for decades to come to reduce the likelihood of this trend continuing. Similar to all enlisted members, Junior enlisted members are disproportionately recruited to serve in the military from communities with higher unemployment (Army Recruiting Command et al. 2022), and receive lower pay for their military service compared to Senior Enlisted, Warrant Officers, and Officers (Defense Finance and Accounting Service 2021). Thus, the finding that junior enlisted members have the highest postdeployment suicide rates may reflect a critical health inequity. Selective and indicated prevention strategies for those at elevated risk are needed, coupled with a public health approach of prioritizing universal strategies for military members and Veterans (Knox and Bossarte 2012; Brenner et al. 2018). This includes lethal means safety planning efforts, particularly regarding firearms, which are the most common mechanism of suicide in this population (Hostetter et al. 2019; Pruitt et al. 2019; Brenner et al. 2018).

## Supplementary Information

**Additional file 1:** Analytic Cohort Creation. Figure/flowchart describing how the final analytic cohort was created.

**Additional file 2:** Sample Characteristics Overall and by Component. Table of demographic and military characteristics for the overall cohort and broken out by military component.

**Additional file 3:** Sample Characteristics by Deployment Group Overall and Within Component. Table of demographic and military characteristics for those whose index deployment was their first (first deployers) and for those whose index deployment was not their first (2+ deployers), within the overall cohort and within each military component.

**Additional file 4:** Female Sample Characteristics. Table of demographic and military characteristics only among female military members, for those whose index deployment was their first (first deployers) and for those whose index deployment was not their first (2+ deployers), within the overall cohort and within each military component.

**Additional file 5:** Component Average Annual Suicide Rates per 100,000 Person Years (October 1, 2007 – December 31, 2018). Table of crude and age-adjusted suicide rates, as well as rate ratios, by military component for the overall cohort and within deployment status (first deployers and 2+ deployers).

**Additional file 6:** Hazard Ratios from Unadjusted and Adjusted Cox Proportional Hazards Models across Military Component. Table of hazard ratios from six Cox proportional hazards models: three unadjusted models, and three models adjusting for demographics, comparing military component within 1) the full cohort, 2) first deployers and 3) 2+ deployers.

**Additional file 7:** Component Average Annual Suicide Rates with 95% CIs. Average annual suicide rates post index deployment per 100,000 person years by military component (years 8-11 combined). Trend analysis annual percent change over time is also presented.

**Additional file 8:** Average Annual Suicide Rates per 100,000 Person Years with Trend Lines by Component. Figure with average annual suicide rates over time post index deployment (per 100,000 person years) by military component with trend lines overlaid.

**Additional file 9:** Hazard Ratios from Cox Proportional Hazards Models across Rank Groups within the Female Cohort. Table of hazard ratios from six Cox proportional hazards models among female military members: three unadjusted models, and three models adjusting for demographics, comparing rank within 1) the full cohort, 2) first deployers and 3) 2+ deployers.

**Additional file 10:** Hazard Ratios from Cox Proportional Hazards Models by Military Component within the Female Cohort. Table of hazard ratios from six Cox proportional hazards models among female military members: three unadjusted models, and three models adjusting for demographics, comparing military component within 1) the full cohort, 2) first deployers and 3) 2+ deployers.

## Data Availability

The Defense Health Agency’s Privacy and Civil Liberties Office provided access to Department of Defense (DoD) data and the VA/DOD Mortality Data Repository (MDR) provided access to National Death Index data. The datasets generated and analyzed during the current study are not publicly available according to our Data Sharing Agreements and are governed by the Defense Health Agency and Veterans Health Affairs, and the MDR, respectively.

## Abbreviations

AD: Active duty
NG: National Guard
RC: Reserve component
DOD: Department of Defense
SUPIC: Substance Use and Psychological Injury Combat study
FY: Fiscal year
MDR: Mortality data repository
NDI: National Death Index
SSN: Social security number
ICD-10: International Classification of Diseases, Tenth Revision
CI: Confidence interval
APC: Annual percent change
HR: Hazard ratio
SAS: Statistical analysis system
