# Publisher Correction to: Time‑dependent suicide rates among Army soldiers returning from an Afghanistan/Iraq deployment, by military rank and component

**DOI:** 10.1186/s40621-023-00432-x

**Published:** 2023-04-24

**Authors:** Rachel Sayko Adams, Jeri E. Forster, Jaimie L. Gradus, Claire A. Hoffmire, Trisha A. Hostetter, Mary Jo Larson, Colin G. Walsh, Lisa A. Brenner

**Affiliations:** 1 Department of Health Law, Policy and Management, Boston University School of Public Health, 715 Albany Street, Boston, MA 02118 USA; 2 Institute for Behavioral Health, The Heller School for Social Policy and Management, Brandeis University, Waltham, MA USA; 3 VHA Rocky Mountain Mental Illness Research Education and Clinical Center, Aurora, CO USA; 4 University of Colorado, Anschutz Medical Campus, Aurora, CO USA; 5 Department of Epidemiology, Boston University School of Public Health, Boston University, Boston, MA USA; 6 Departments of Biomedical Informatics, Medicine, and Psychiatry, Vanderbilt University Medical Center, Nashville, TN USA

**Correction to: Injury Epidemiology 9:46 (2022)** https://doi.org/10.1186/s40621-022-00410-9

Following publication of the original article (Adams et al. 2022), the authors identified that the author group were indicated twice.

The publisher apologizes for the inconvenience caused.

The author group has been updated above and the original article (Adams et al. 2022) has been corrected.
