# Supplementary figures and images for: Time-dependent suicide rates among Army soldiers returning from an Afghanistan/Iraq deployment, by military rank and component
Source: Inj Epidemiol. 2022 Dec 23;9:46. doi: 10.1186/s40621-022-00410-9 (PMC9783392; doi:10.1186/s40621-022-00410-9)

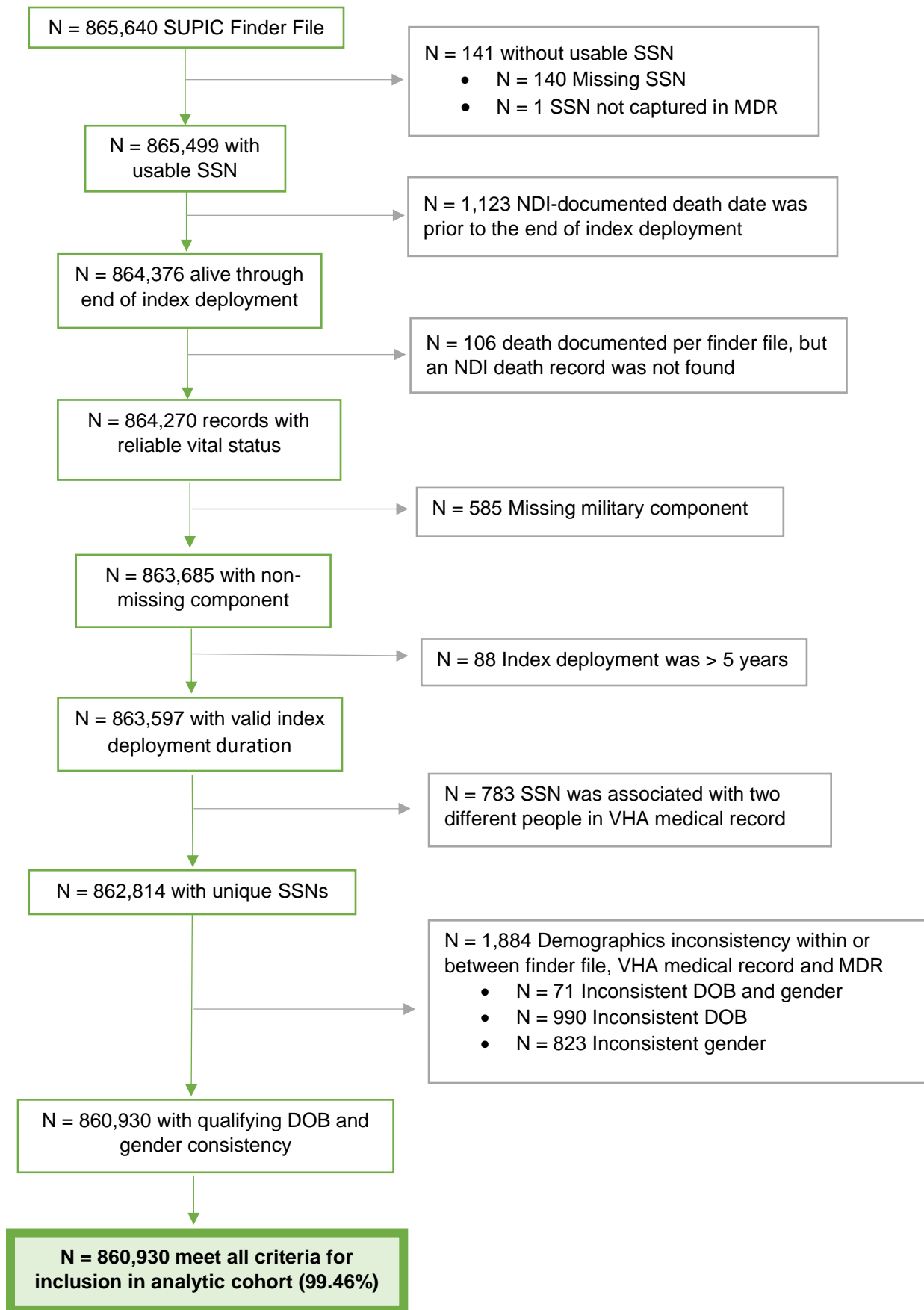

Supplement: Supplementary file 1 — Additional file 1: Analytic Cohort Creation. Figure/flowchart describing how the final analytic cohort was created. [file 40621_2022_410_MOESM1_ESM.pdf]

Average Annual Suicide Rate per 100,000 Person Years

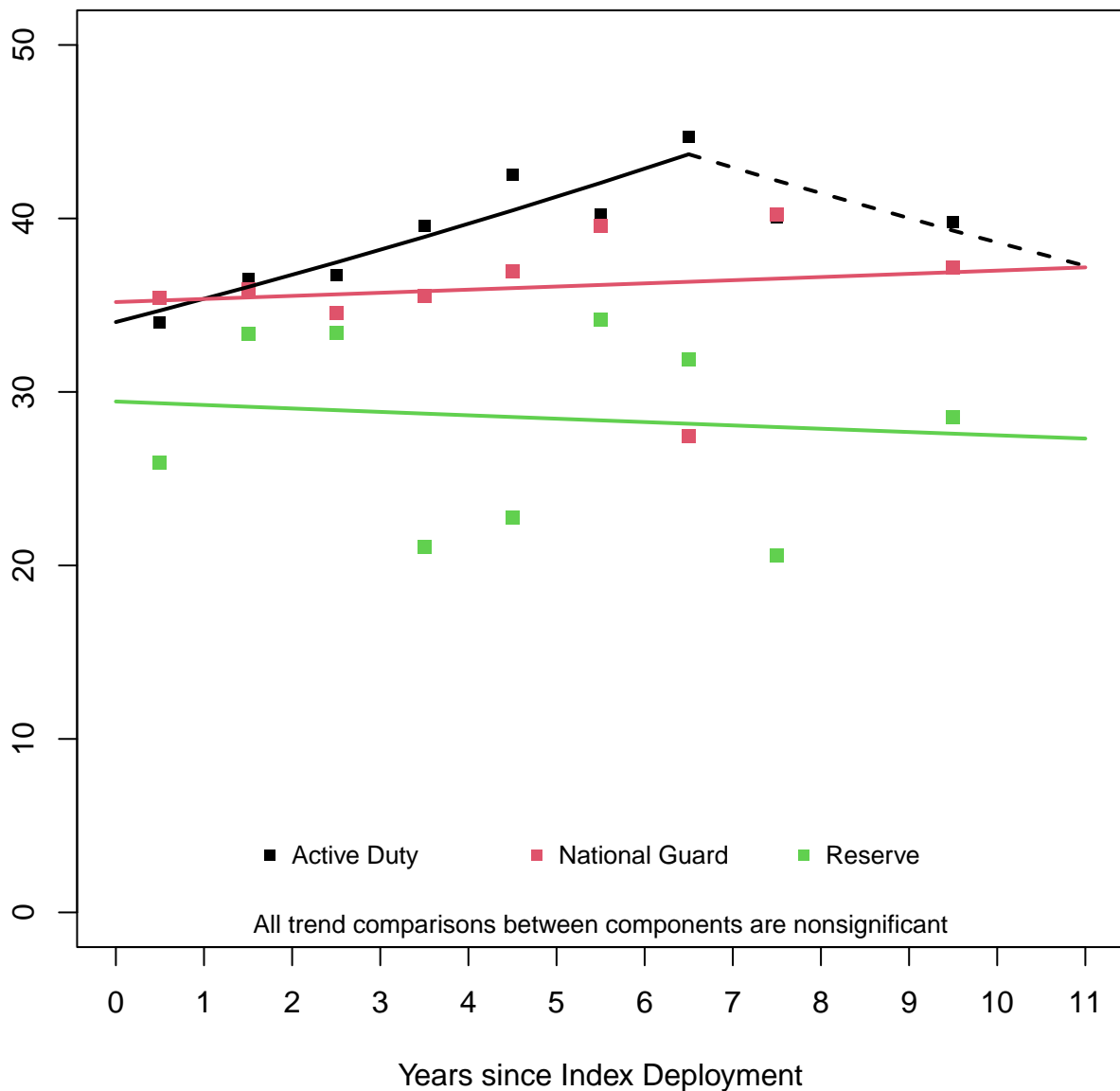

Supplement: Supplementary file 8 — Additional file 8: Average Annual Suicide Rates per 100,000 Person Years with Trend Lines by Component. Figure with average annual suicide rates over time post index deployment (per 100,000 person years) by military component with trend lines overlaid. [file 40621_2022_410_MOESM8_ESM.pdf]
