# Supplementary material for: Time-dependent suicide rates among Army soldiers returning from an Afghanistan/Iraq deployment, by military rank and component
Source: Inj Epidemiol. 2022 Dec 23;9:46. doi: 10.1186/s40621-022-00410-9 (PMC9783392; doi:10.1186/s40621-022-00410-9)
Supplement: Supplementary file 2 — Additional file 2: Sample Characteristics Overall and by Component. Table of demographic and military characteristics for the overall cohort and broken out by military component. [file 40621_2022_410_MOESM2_ESM.docx]

Additional File 2. Sample Characteristics Overall and by Component

|  | **Overall**  **N (%)** | **Active Duty**  **N (%)** | **National Guard**  **N (%)** | **Reserves**  **N (%)** |
| --- | --- | --- | --- | --- |
| **Total** | 860,930 (100%) | 573,531 (66.6%) | 206,332 (24.0%) | 81,067 (9.4%) |
| **Rank Group** |  |  |  |  |
| Junior Enlisted (E1-E4) | 413,463 (48.0%) | 291,274 (50.8%) | 94,495 (45.8%) | 27,694 (34.2%) |
| Senior Enlisted (E5-E9)/Warrant Officer | 339,205 (39.4%) | 196,614 (34.3%) | 86,609 (42.0%) | 36,108 (44.5%) |
| Officer | 108,257 (12.6%) | 72,241 (12.6%) | 20,475 (9.9%) | 15,541 (19.2%) |
| Missing | 5 (0%) | 2 (0%) | 2 (0%) | 1 (0%) |
| **Age Category at**  **End of Index Deployment** |  |  |  |  |
| 18-24 | 320,548 (37.2%) | 235,117 (41.0%) | 64,651 (31.3%) | 20,780 (25.6%) |
| 25-29 | 217,275 (25.2%) | 152,918 (26.7%) | 46,014 (22.3%) | 18,343 (22.6%) |
| 30-34 | 117,585 (13.7%) | 79,606 (13.9%) | 27,686 (13.4%) | 10,293 (12.7%) |
| 35-39 | 92,002 (10.7%) | 58,442 (10.2%) | 24,440 (11.8%) | 9120 (11.3%) |
| 40+ | 113,520 (13.2%) | 47,448 (8.3%) | 43,541 (21.1%) | 22,531 (27.8%) |
| **Gender** |  |  |  |  |
| Male | 766,489 (89%) | 512,087 (89.3%) | 186,378 (90.3%) | 68,024 (83.9%) |
| Female | 94,441 (11%) | 61,444 (10.7%) | 19,954 (9.7%) | 13,043 (16.1%) |
| **Race/Ethnicity** |  |  |  |  |
| American Indian/Alaskan Native | 7918 (0.9%) | 5186 (0.9%) | 1989 (1.0%) | 743 (0.9%) |
| Asian or Pacific Islander | 68,699 (8%) | 58,887 (10.3%) | 5877 (2.9%) | 3935 (4.9%) |
| Black non-Hispanic | 143,350 (16.6%) | 103,092 (18%) | 26,160 (12.7%) | 14,098 (17.4%) |
| White non-Hispanic | 539,434 (62.7%) | 333,404 (58.1%) | 154,338 (74.8%) | 51,692 (63.8%) |
| Hispanic | 91,365 (10.6%) | 65,475 (11.4%) | 15,832 (7.7%) | 10,058 (12.4%) |
| Other | 7838 (0.9%) | 6203 (1.1%) | 1371 (0.7%) | 264 (0.3%) |
| Unknown/Missing | 2326 (0.3%) | 1284 (0.2%) | 765 (0.4%) | 277 (0.3%) |
| **Index Deployment Group** |  |  |  |  |
| First Deployers | 598,335 (69.5%) | 386,283 (67.4%) | 152,539 (73.9%) | 59,513 (73.4%) |
| 2+ Deployers | 262,595 (30.5%) | 187,248 (32.6%) | 53,793 (26.1%) | 21,554 (26.6%) |
| **Fiscal Year of Return from Index Deployment** |  |  |  |  |
| 2008-09 | 316,420 (36.8%) | 223,354 (38.9%) | 67,808 (32.9%) | 25,258 (31.2%) |
| 2010-11 | 326,101 (37.9%) | 209,520 (36.5%) | 83,977 40.7%) | 32,604 (40.2%) |
| 2012-14 | 218,409 (25.4%) | 140,657 (24.5%) | 54,547 (26.4%) | 23,205 (28.6%) |
