# Supplementary material for: Time-dependent suicide rates among Army soldiers returning from an Afghanistan/Iraq deployment, by military rank and component
Source: Inj Epidemiol. 2022 Dec 23;9:46. doi: 10.1186/s40621-022-00410-9 (PMC9783392; doi:10.1186/s40621-022-00410-9)
Supplement: Supplementary file 3 — Additional file 3: Sample Characteristics by Deployment Group Overall and Within Component. Table of demographic and military characteristics for those whose index deployment was their first (first deployers) and for those whose index deployment was not their first (2+ deployers), within the overall cohort and within each military component. [file 40621_2022_410_MOESM3_ESM.docx]

Additional File 3. Sample Characteristics by Deployment Group Overall and Within Component

|  | **Full Cohort** | | **Active Duty** | | **National Guard** | | **Reserve** | |
| --- | --- | --- | --- | --- | --- | --- | --- | --- |
|  | **First Deployers** | **2+ Deployers** | **First Deployers** | **2+ Deployers** | **First Deployers** | **2+ Deployers** | **First Deployers** | **2+ Deployers** |
| **Rank Group** |  |  |  |  |  |  |  |  |
| Junior Enlisted (E1-E4) | 366,097  (61.2%) | 47,366  (18.0%) | 255,810  (66.2%) | 35,464  (18.9%) | 85,006  (55.7%) | 9489  (17.6%) | 25,281  (42.5%) | 2413  (11.2%) |
| Senior Enlisted (E5-E9)/Warrant Officer | 160,915  (26.9%) | 178,290  (67.9%) | 83,653  (21.7%) | 126,361 (67.5%) | 53,478 (35.1%) | 37,882 (70.4%) | 23,784 (40.0%) | 14,047 (65.2%) |
| Officer | 71,319  (11.9%) | 36,938  (14.0%) | 46,818  (12.1%) | 25,423  (13.6%) | 14,054  (9.2%) | 6421 (12.0%) | 10,447  (17.6%) | 5094  (23.6%) |
| Missing | 4 (0%) | 1 (0%) | 2 (0%) | 0 (0%) | 1 (0%) | 1 (0%) | 1(0%) | 0 (0%) |
| **Age Category** |  |  |  |  |  |  |  |  |
| 18-24 | 282,307  (47.1%) | 38,241  14.6%) | 202,884  (52.5%) | 32,233  (17.2%) | 60,110  (39.4%) | 4541  (8.4%) | 19,313  (32.5%) | 1467  (6.8%) |
| 25-29 | 138,684  (23.2%) | 78,591  (29.9%) | 93,762  (24.3%) | 59,156  (31.6%) | 32,296  (21.2%) | 13,718  (25.5%) | 12,626  (21.2%) | 5717  (26.5%) |
| 30-34 | 64,306  (10.8%) | 53,279  (20.3%) | 39,752  (10.3%) | 39,854  (21.3%) | 17,779  (11.7%) | 9907  (18.4%) | 6775  (11.4%) | 3518  (16.3%) |
| 35-39 | 49,060  (8.2%) | 42,942  (16.4%) | 27,386  (7.1%) | 31,056  (16.6%) | 15,489  (10.2%) | 8951  (16.6%) | 6185  (10.4%) | 2935  (13.6%) |
| 40+ | 63,978  (10.7%) | 49,542  (18.9%) | 22,499  (5.8%) | 24,949  (13.3%) | 26,865  (17.6%) | 16,676  (31.0%) | 14,614  (24.6%) | 7917  (36.7%) |
| **Gender** |  |  |  |  |  |  |  |  |
| Male | 524,621 (87.7%) | 241,868  (92.1%) | 340,051  (88.0%) | 172,036  (91.9%) | 135,523  (88.8%) | 50,855  (94.5%) | 49,047  (82.4%) | 18,977  (88.0%) |
| Female | 73,714  (12.3%) | 20,727  (7.9%) | 46,232  (12.0%) | 15,212  (8.1%) | 17,016  (11.2%) | 2938  (5.5%) | 10,466  (17.6%) | 2577  (12.0%) |
| **Race/Ethnicity** |  |  |  |  |  |  |  |  |
| American Indian/Alaskan Native | 5529  (0.9%) | 2389  (0.9%) | 3502  (0.9%) | 1684  (0.9%) | 1474  (1.0%) | 515  (1.0%) | 553  (0.9%) | 190  (0.9%) |
| Asian or Pacific Islander | 46,257  (7.7%) | 22,442  (8.6%) | 39,209  (10.2%) | 19,678  (10.5%) | 4136  (2.7%) | 1741  (3.2%) | 2912  (4.9%) | 1023  (4.8%) |
| Black non-Hispanic | 96,224  (16.1%) | 47,126  (18.0%) | 65,587  (17.0%) | 37,505  (20.0%) | 20,013  (13.1%) | 6147  (11.4%) | 10,624  (17.9%) | 3474  (16.1%) |
| White non-Hispanic | 380,734  (63.6%) | 158,700  (60.4%) | 229,802  (59.5%) | 103,602  (55.3%) | 113,303  (74.3%) | 41,035  (76.3%) | 37,629  (63.2%) | 14,063  (65.3%) |
| Hispanic | 63,779  (10.7%) | 27,586  (10.5%) | 44,229  (11.5%) | 21,246  (11.4%) | 12,133  (8.0%) | 3699  (6.9%) | 7417  (12.5%) | 2641  (12.3%) |
| Other | 4105  (0.7%) | 3733  (1.4%) | 3042  (0.8%) | 3161  (1.7%) | 901  (0.6%) | 470  (0.9%) | 162  (0.3%) | 102  (0.5%) |
| Unknown/Missing | 1707  (0.3%) | 619  (0.2%) | 912  (0.2%) | 372  (0.2%) | 579  (0.4%) | 186  (0.4%) | 216  (0.4%) | 61  (0.3%) |
| **Fiscal Year of Return from Index Deployment** |  |  |  |  |  |  |  |  |
| FY2008-FY2009 | 199,139  (33.3%) | 117,281  (44.7%) | 128,896  (33.4%) | 94,458  (50.5%) | 51,396  (33.7%) | 16,412  (30.5%) | 18,847  (31.7%) | 6411  (29.7%) |
| FY2010-FY2011 | 209,532  (35.0%) | 116,569  (44.4%) | 131,143  (34.0%) | 78,377  (41.9%) | 56,736  (37.2%) | 27,241  (50.6%) | 21,653  (36.4%) | 10,951  (50.8%) |
| FY2012-FY2014 | 189,664  (31.7%) | 28,745  (11.0%) | 126,244  (32.7%) | 14,413  (7.7%) | 44,407  (29.1%) | 10,140  (18.9%) | 19,013  (32.0%) | 4192  (19.5%) |
