# Supplementary material for: Time-dependent suicide rates among Army soldiers returning from an Afghanistan/Iraq deployment, by military rank and component
Source: Inj Epidemiol. 2022 Dec 23;9:46. doi: 10.1186/s40621-022-00410-9 (PMC9783392; doi:10.1186/s40621-022-00410-9)
Supplement: Supplementary file 4 — Additional file 4: Female Sample Characteristics. Table of demographic and military characteristics only among female military members, for those whose index deployment was their first (first deployers) and for those whose index deployment was not their first (2+ deployers), within the overall cohort and within each military component. [file 40621_2022_410_MOESM4_ESM.docx]

Additional File 4. Female Sample Characteristics

|  | **Overall** | | **Active Duty** | | **National Guard** | | **Reserve** | |
| --- | --- | --- | --- | --- | --- | --- | --- | --- |
|  | First Deployers | 2+ Deployers | First Deployers | 2+ Deployers | First Deployers | 2+ Deployers | First Deployers | 2+ Deployers |
| **Age** |  |  |  |  |  |  |  |  |
| 18-24 | 32,706  (44.4%) | 3262  (15.7%) | 21,579  (46.7%) | 2726  (17.9%) | 7527  (44.2%) | 327  (11.1%) | 3600  (34.4%) | 209  (8.1%) |
| 25-29 | 17,914  (24.3%) | 6672  (32.2%) | 11,748  (25.4%) | 5025  (33.0%) | 3895  (22.9%) | 929  (31.6%) | 2271  (21.7%) | 718  (27.9%) |
| 30-34 | 8717  (11.8%) | 4133  (19.9%) | 5638  (12.2%) | 3094  (20.3%) | 1915  (11.3%) | 583  (19.8%) | 1164  (11.1%) | 456  (17.7%) |
| 35-39 | 6183  (8.4%) | 3053  (14.7%) | 3831  (8.3%) | 2366  (15.6%) | 1366  (8.0%) | 385  (13.1%) | 986  (9.4%) | 302  (11.7%) |
| 40+ | 8194  (11.1%) | 3607  (17.4%) | 3436  (7.4%) | 2001  (13.2%) | 2313  (13.6%) | 714  (24.3%) | 2445  (23.4%) | 892  (34.6%) |
| **Race** |  |  |  |  |  |  |  |  |
| American Indian/Alaskan Native | 937  (1.3%) | 284  (1.4%) | 617  (1.3%) | 223  (1.5%) | 201  (1.2%) | 37  (1.3%) | 119  (1.1%) | 24  (0.9%) |
| Asian or Pacific Islander | 6018  (8.2%) | 1833  (8.8%) | 4947  (10.7%) | 1572  (10.3%) | 543  (3.2%) | 129  (4.4%) | 528  (5.0%) | 132  (5.1%) |
| Black non-Hispanic | 22,359  (30.3%) | 7633  (36.8%) | 15,271  (33.0%) | 6168  (40.6%) | 3953  (23.2%) | 647  (22.0%) | 3135  (30.0%) | 818  (31.7%) |
| White non-Hispanic | 34,206  (46.4%) | 8034  (38.8%) | 18,587  (40.2%) | 4926  (32.4%) | 10,442  (61.4%) | 1844  (62.8%) | 5177  (49.5%) | 1264  (49.1%) |
| Hispanic | 9081  (12.3%) | 2459  (11.9%) | 6035  (13.1%) | 1923  (12.6%) | 1612  (9.5%) | 221  (7.5%) | 1434  (13.7%) | 315  (12.2%) |
| Other | 679  (0.9%) | 365  (1.8%) | 501  (1.1%) | 316  (2.1%) | 145  (0.9%) | 38  (1.3%) | 33  (0.3%) | 11  (0.4%) |
| Unknown  / Missing | 434  (0.6%) | 119  (0.6%) | 274  (0.6%) | 84  (0.6%) | 120  (0.7%) | 22  (0.8%) | 40  (0.4%) | 13  (0.5%) |
| **Rank** |  |  |  |  |  |  |  |  |
| Junior Enlisted (E1-E4) | 40,639  (55.1%) | 3619  (8.2%) | 26,691  (57.7%) | 2844  (18.7%) | 9596  (56.4%) | 511  (17.4%) | 4352  (41.6%) | 264  (10.2%) |
| Senior Enlisted (E5-E9) /Warrant Officer | 21,656  (29.4%) | 13,523  (65.2%) | 11,592  (25.1%) | 9823  (64.6%) | 5875  (34.5%) | 2020  (68.8%) | 4189  (40.0%) | 1680  (65.2%) |
| Officer | 11,419  (15.5%) | 3585  (17.3%) | 7949  (20.2%) | 2545  (16.7%) | 1545  (9.1%) | 407  (13.9%) | 1925  (18.4%) | 633  (24.6%) |
| **FY of Return from Index Deployment** |  |  |  |  |  |  |  |  |
| FY2008-FY2009 | 23,417  (31.8%) | 8686  (41.9%) | 15,540  (33.6%) | 7116  (46.8%) | 4669  (27.4%) | 769  (26.2%) | 3208  (30.7%) | 801  (31.1%) |
| FY2010-FY2011 | 26,166  (35.5%) | 9148  (44.1%) | 16,007  (34.6%) | 6510  (42.8%) | 6554  (38.5%) | 1433  (48.8%) | 3605  (34.4%) | 1205  (46.8%) |
| FY2012-FY2014 | 24,131  (32.7%) | 2893  (14.0%) | 14,685  (31.8%) | 1586  (10.4%) | 5793  (34.0%) | 736  (25.1%) | 3653  (34.9%) | 571  (22.2%) |
