# Supplementary material for: Time-dependent suicide rates among Army soldiers returning from an Afghanistan/Iraq deployment, by military rank and component
Source: Inj Epidemiol. 2022 Dec 23;9:46. doi: 10.1186/s40621-022-00410-9 (PMC9783392; doi:10.1186/s40621-022-00410-9)
Supplement: Supplementary file 5 — Additional file 5: Component Average Annual Suicide Rates per 100,000 Person Years (October 1, 2007 – December 31, 2018). Table of crude and age-adjusted suicide rates, as well as rate ratios, by military component for the overall cohort and within deployment status (first deployers and 2+ deployers). [file 40621_2022_410_MOESM5_ESM.docx]

Additional File 5. Component Average Annual Suicide Rates per 100,000 Person Years (October 1, 2007 – December 31, 2018)

| **Component** | Crude Rate  (95% CI) | Age-adjusted Rate^a^  (95% CI) | Rate Ratio  (95% CI) |
| --- | --- | --- | --- |
| Active Duty | 389.18  (37.44, 40.98) | 26.12  (23.64, 28.88) | Ref |
| National Guard | 35.80  (33.01, 38.75) | 26.79  (23.96, 29.94) | 1.03  (0.88, 1.19) |
| Reserve | 28.09  (24.20, 32.43) | 22.36  (18.55, 26.87) | 0.86  (0.69, 1.06) |
| **First Deployers** |  |  |  |
| Active Duty | 42.73  (40.47, 45.08) | 27.45  (23.78, 31.73) | Ref |
| National Guard | 37.23  (33.93, 40.76) | 27.15  (23.59, 31.25) | 0.99  (0.81, 1.21) |
| Reserve | 30.74  (25.98, 36.11) | 23.94  (19.15, 29.82) | 0.87  (0.67, 1.14) |
| **2+ Deployers** |  |  |  |
| Active Duty | 32.64  (29.98, 35.47) | 27.32^b^  (24.68, 30.20) | Ref |
| National Guard | 31.83  (26.84, 37.47) | 30.07^b^  (25.14, 35.72) | 1.10  (0.90, 1.35) |
| Reserve | 21.05  (14.89, 28.88) | 18.55^b^  (12.88, 26.04) | **0.68**  **(0.46, 0.97)** |

^a^Age-adjusted rates based on the following age categories: 18-24, 25-29, 30-34, 35-39, 40+

^b^Age-adjusted rates based on age categories 18-29 and 30+

Boldface indicates statistical significance (p<0.05).
