# Supplementary material for: Time-dependent suicide rates among Army soldiers returning from an Afghanistan/Iraq deployment, by military rank and component
Source: Inj Epidemiol. 2022 Dec 23;9:46. doi: 10.1186/s40621-022-00410-9 (PMC9783392; doi:10.1186/s40621-022-00410-9)
Supplement: Supplementary file 6 — Additional file 6: Hazard Ratios from Unadjusted and Adjusted Cox Proportional Hazards Models across Military Component. Table of hazard ratios from six Cox proportional hazards models: three unadjusted models, and three models adjusting for demographics, comparing military component within 1) the full cohort, 2) first deployers and 3) 2+ deployers. [file 40621_2022_410_MOESM6_ESM.docx]

Additional File 6. Hazard Ratios from Unadjusted and Adjusted Cox Proportional Hazard Models

across Military Component

|  | Unadjusted model | Adjusting for  Demographics^a^ |
| --- | --- | --- |
| Full Cohort | Hazard Ratio  (95% CI) | Hazard Ratio  (95% CI) |
| Active Duty vs National Guard | **1.10**  **(1.00, 1.20)** | 1.03  (0.94, 1.13) |
| Active Duty vs Reserve | **1.39**  **(1.20, 1.62)** | 1.15  (0.99, 1.34) |
| National Guard vs Reserve | **1.27**  **(1.08, 1.50)** | 1.12  (0.95, 1.32) |
| First Deployers |  |  |
| Active Duty vs National Guard | **1.15**  **(1.03, 1.28)** | 1.07  (0.96, 1.19) |
| Active Duty vs Reserve | **1.39**  **(1.17, 1.65)** | 1.13  (0.95, 1.34) |
| National Guard vs Reserve | **1.21**  **(1.01, 1.46)** | 1.06  (0.88, 1.27) |
| 2+ Deployers |  |  |
| Active Duty vs National Guard | 1.03  (0.86, 1.24) | 0.95  (0.78, 1.15) |
| Active Duty vs Reserve | **1.55**  **(1.12, 2.16)** | 1.30  (0.93, 1.81) |
| National Guard vs Reserve | **1.51**  **(1.06, 2.16)** | 1.37  (0.96, 1.96) |

^a^Adjusted for gender, age category (18-24, 25-29, 30-34, 35-39, 40+), race/ethnicity (American Indian/Alaskan Native, Asian or Pacific Islander, Black non-Hispanic, White non-Hispanic, Hispanic, other, unknown/missing) and Fiscal Year of return from index deployment grouped as 2008-09, 2010-11, and 2012-14.

Boldface indicates statistical significance (p<0.05).
