# Supplementary material for: Time-dependent suicide rates among Army soldiers returning from an Afghanistan/Iraq deployment, by military rank and component
Source: Inj Epidemiol. 2022 Dec 23;9:46. doi: 10.1186/s40621-022-00410-9 (PMC9783392; doi:10.1186/s40621-022-00410-9)
Supplement: Supplementary file 7 — Additional file 7: Component Average Annual Suicide Rates with 95% CIs. Average annual suicide rates post index deployment per 100,000 person years by military component (years 8-11 combined). Trend analysis annual percent change over time is also presented. [file 40621_2022_410_MOESM7_ESM.docx]

Additional File 7. Component Average Annual Suicide Rates with 95% CIs

|  | Years Since End of Index Deployment | | | | | | | | |  |
| --- | --- | --- | --- | --- | --- | --- | --- | --- | --- | --- |
|  | 0-1 Year | 1-2 Years | 2-3 Years | 3-4 Years | 4-5 Years | 5-6 Years | 6-7 Years | 7-8 Years | 8-11 Years | APC^b^  (95% CI) |
| Active Duty | 34.03  (29.3, 38.8) | 36.51  (31.6, 41.5) | 36.74  (31.8, 41.7) | 39.59  (34.4, 44.8) | 42.50  (37.1, 47.9) | 40.20  (34.8, 45.6) | 44.68  (38.7, 50.7) | 40.04  (33.9, 46.2) | 39.79  (34.6, 45.0) | Years 0-6.5:  **3.9 (1.3, 6.6)**  Years 6.5-11:  -3.5 (-10.9, 4.5) |
| National  Guard | 35.40  (27.3, 43.5) | 35.93  (27.7, 44.1) | 34.53  (26.5, 42.6) | 35.54  (27.4, 43.7) | 36.93  (28.6, 45.3) | 39.57  (30.6, 48.5) | 27.46  (19.6, 35.3) | 40.24  (29.8, 50.7) | 37.15  (28.2, 46.1) | 0.5 (-2.4, 3.5) |
| Reserve | 25.92  (14.8, 37.0) | 33.35  (20.8, 46.0) | 33.39  (20.8, 46.0) | 21.05  (11.0, 31.0) | 22.76  (12.2, 33.3) | 34.18  (20.8, 47.6) | 31.85  (18.2, 45.5) | 20.55^a^  (8.41, 32.7) | 28.55  (15.7, 41.4) | -0.7 (-6.5, 5.5) |

*Note.* Suicide rates are per 100,000 person years.

^a^Cell size <16, unreliable; ^b^Annual Percent Change estimated using trend analysis

Boldface indicates statistical significance (p<0.05).
