# Supplementary material for: Time-dependent suicide rates among Army soldiers returning from an Afghanistan/Iraq deployment, by military rank and component
Source: Inj Epidemiol. 2022 Dec 23;9:46. doi: 10.1186/s40621-022-00410-9 (PMC9783392; doi:10.1186/s40621-022-00410-9)
Supplement: Supplementary file 9 — Additional file 9: Hazard Ratios from Cox Proportional Hazards Models across Rank Groups within the Female Cohort. Table of hazard ratios from six Cox proportional hazards models among female military members: three unadjusted models, and three models adjusting for demographics, comparing rank within 1) the full cohort, 2) first deployers and 3) 2+ deployers. [file 40621_2022_410_MOESM9_ESM.docx]

Additional File 9. Hazard Ratios from Cox Proportional Hazard Models across Rank Groups within the Female Cohort

|  | Unadjusted model | Adjusting for  Demographics^a^ |
| --- | --- | --- |
| Full Female Cohort | Hazard Ratio  (95% CI) | Hazard Ratio  (95% CI) |
| Junior Enlisted (E1-E4) vs Senior Enlisted (E5-E9) /Warrant Officer | 1.13  (0.77, 1.67) | 0.84  (0.54, 1.32) |
| Junior Enlisted (E1-E4) vs Officer | **2.05**  **(1.05, 4.00)** | 1.71  (0.83, 3.53) |
| Senior Enlisted (E5-E9) /Warrant Officer vs Officer | 1.81  (0.91, 3.60) | **2.03**  **(1.02, 4.05)** |
| First Deployers |  |  |
| Junior Enlisted (E1-E4) vs Senior Enlisted (E5-E9) /Warrant Officer | 1.05  (0.67, 1.63) | 0.86  (0.52, 1.42) |
| Junior Enlisted (E1-E4) vs Officer | 1.98  (0.94, 4.15) | 1.89  (0.86, 4.18) |
| Senior Enlisted (E5-E9) /Warrant Officer vs Officer | 1.89  (0.87, 4.12) | **2.21**  **(1.01, 4.84)** |
| 2+ Deployers |  |  |
| Junior Enlisted (E1-E4) vs Senior Enlisted (E5-E9) /Warrant Officer | 0.91  (0.30, 2.75) | 0.63  (0.19, 2.03) |
| Junior Enlisted (E1-E4) vs Officer | 1.70  (0.31, 9.31) | 1.03  (0.16, 6.69) |
| Senior Enlisted (E5-E9) /Warrant Officer vs Officer | 1.86  (0.43, 8.15) | 1.65  (0.36, 7.61) |

^a^Adjusted for gender, age category (18-24, 25-29, 30-34, 35-39, 40+), race/ethnicity (Black, White, Hispanic, Other), and Fiscal Year of return from index deployment grouped as 2008-09, 2010-11, and 2012-14.

Boldface indicates statistical significance (p<0.05).
