# Supplementary material for: Time-dependent suicide rates among Army soldiers returning from an Afghanistan/Iraq deployment, by military rank and component
Source: Inj Epidemiol. 2022 Dec 23;9:46. doi: 10.1186/s40621-022-00410-9 (PMC9783392; doi:10.1186/s40621-022-00410-9)
Supplement: Supplementary file 10 — Additional file 10: Hazard Ratios from Cox Proportional Hazards Models by Military Component within the Female Cohort. Table of hazard ratios from six Cox proportional hazards models among female military members: three unadjusted models, and three models adjusting for demographics, comparing military component within 1) the full cohort, 2) first deployers and 3) 2+ deployers. [file 40621_2022_410_MOESM10_ESM.docx]

Additional File 10. Hazard Ratios from Cox Proportional Hazard Models by Military Component within the Female Cohort

|  | Unadjusted model | Adjusting for  Demographics^a^ |
| --- | --- | --- |
| Full Female Cohort | Hazard Ratio  (95% CI) | Hazard Ratio  (95% CI) |
| Active Duty vs National Guard/Reserve | 0.77  (0.53, 1.11) | 0.82  (0.56, 1.20) |
| National Guard /Reserve vs Active Duty | 1.31  (0.90, 1.90) | 1.22  (0.83, 1.80) |
| First Deployers |  |  |
| Active Duty vs National Guard/Reserve | 0.85  (0.57, 1.29) | 0.90  (0.59, 1.37) |
| National Guard /Reserve vs Active Duty | 1.17  (0.78, 1.77) | 1.12  (0.73, 1.71) |
| 2+ Deployers |  |  |
| Active Duty vs National Guard/Reserve | 0.50  (0.21, 1.22) | 0.46  (0.18, 1.18) |
| National Guard /Reserve vs Active Duty | 1.98  (0.82, 4.79) | 2.16  (0.85, 5.49) |

^a^Adjusted for gender, age category (18-24, 25-29, 30-34, 35-39, 40+), race/ethnicity (Black, White, Hispanic, Other), and Fiscal Year of return from index deployment grouped as 2008-09, 2010-11, and 2012-14.
